# A post-trial follow-up study of pentosan polysulfate monotherapy on preventing recurrent urinary tract infection in women

**DOI:** 10.1038/s41598-022-21100-y

**Published:** 2022-10-06

**Authors:** Yuan-Ju Lee, Shang-Jen Chang, Hsiu-Ying Lin, En Meng, Jeff S. Chueh, Chi-Shin Tseng

**Affiliations:** 1 Present Address: Department of Urology, National Taiwan University Hospital, National Taiwan University, No. 7, Chung-Shan South Road, Taipei, 100 Taiwan; 2 Department of Urology, Taipei Tzu Chi Hospital, New Taipei City, Taiwan; 3 Division of Urology, Department of Surgery, Tri-Service General Hospital, National Defense Medical Center, Taipei, Taiwan; 4 Graduate Institute of Clinical Medicine, National Taiwan University College of Medicine, Taipei, Taiwan

**Keywords:** Urinary tract infection, Quality of life

## Abstract

For women with recurrent urinary tract infection (UTI), previous U101 study has shown that pentosan polysulfate sodium (PPS) monotherapy for 16 weeks significantly reduced UTI episodes in the treatment group throughout the trial period. In this follow-up study, we aimed to assess whether the effects of PPS would last after completion of the trial to prevent recurrent UTIs. Conducted from 2018 to 2019, the U101 study was a multicenter, prospective, phase 2a, randomized trial, enrolling women with recurrent UTI to study the effects of a 16-week oral PPS monotherapy. After approximately two years, the follow-up was conducted by phone interview, obtaining data including self-reported UTI events, quality of life questionnaire, and adverse events. The primary endpoint of follow-up study was UTI recurrence-free survival and the secondary endpoints were quality of life and adverse events. Approximately two years after completion of the trial, the rate of recurrent UTI was 25% (3 of the 12 patients) in the PPS group and 85.7% (12 of the 14 patients) in the control group. Over the entire follow-up period, the UTI recurrence-free survival was significantly better in the PPS group than in the control group (log-rank test *p* < 0.001). The quality of life at two years was significantly improved in the PPS when compared to the control group (91.7 vs. 77.5, *p* < 0.001). No late adverse event was observed after cessation of the treatment. In this study, sixteen weeks of PPS monotherapy in women with recurrent UTI significantly reduced the numbers of recurrent UTI episodes during the 2-year follow-up.

## Introduction

The lifetime risk of a woman developing a UTI is around 30–50%^1,2^, and roughly 21–53% experience symptomatic recurrent infections within a year after completing the treatment^3,4^. Women with recurrent UTI, defined as at least two episodes of UTI in six months or three episodes in twelve months, are frequently exposed to antibiotics which might increase the risk of antimicrobial resistance and cause side effects from antibiotics^5,6^. Several guidelines and reviews have provided prevention strategies based on the best evidence for general or specific populations of women with recurrent UTI^7,8^. However, antibiotic prophylaxis for recurrent UTIs remains the most effective method, outweighing other nonantibiotic alternatives with unsatisfying effectiveness, such as daily cranberry pills, daily estrogen therapy, and acupuncture^8–10^.

Previously, we had published the outcome of a new nonantibiotic management for recurrent UTI in a multi-center Phase 2a, randomized trial using PPS as monotherapy, showing great efficacy of PPS in preventing recurrent UTI during the 16-week trial period^11^. In the control arm, the median time to first confirmed recurrent UTI was 85.5 days and the overall UTI recurrence rate was 64% at the end of the trial. The high protection rate of PPS in the treatment arm led to early termination of patient enrollment of the trial and quick launching of a phase 3 randomized, double-blind, placebo-controlled study. Notwithstanding the observed in-trial benefits of PPS monotherapy, an extended follow-up study of the previous phase 2a U101 trial was planned to investigate the long-term protective effects of the treatment and the quality of life (QoL) of participants.

## Methods

### Trial design

The details of U101 study, including patients, methods, design, and execution have previously been published^11^. The protocol was approved by the institutional review boards at National Taiwan University Hospital (IRB# 201802019MSC), Taipei Tzu Chi Hospital (IRB# 07-FS01-013), and Tri-Service General Hospital (IRB# 1-106-01-035) and conducted in accordance with the principles of Good Clinical Practices and the ethical principles of the Declaration of Helsinki. Written informed consent was obtained from patients before inclusion. The intervention drugs of oral PPS (Urosan®) and laboratory tests were sponsored by TCM Biotech International Corporation (Taipei, Taiwan).

**Figure 1:**
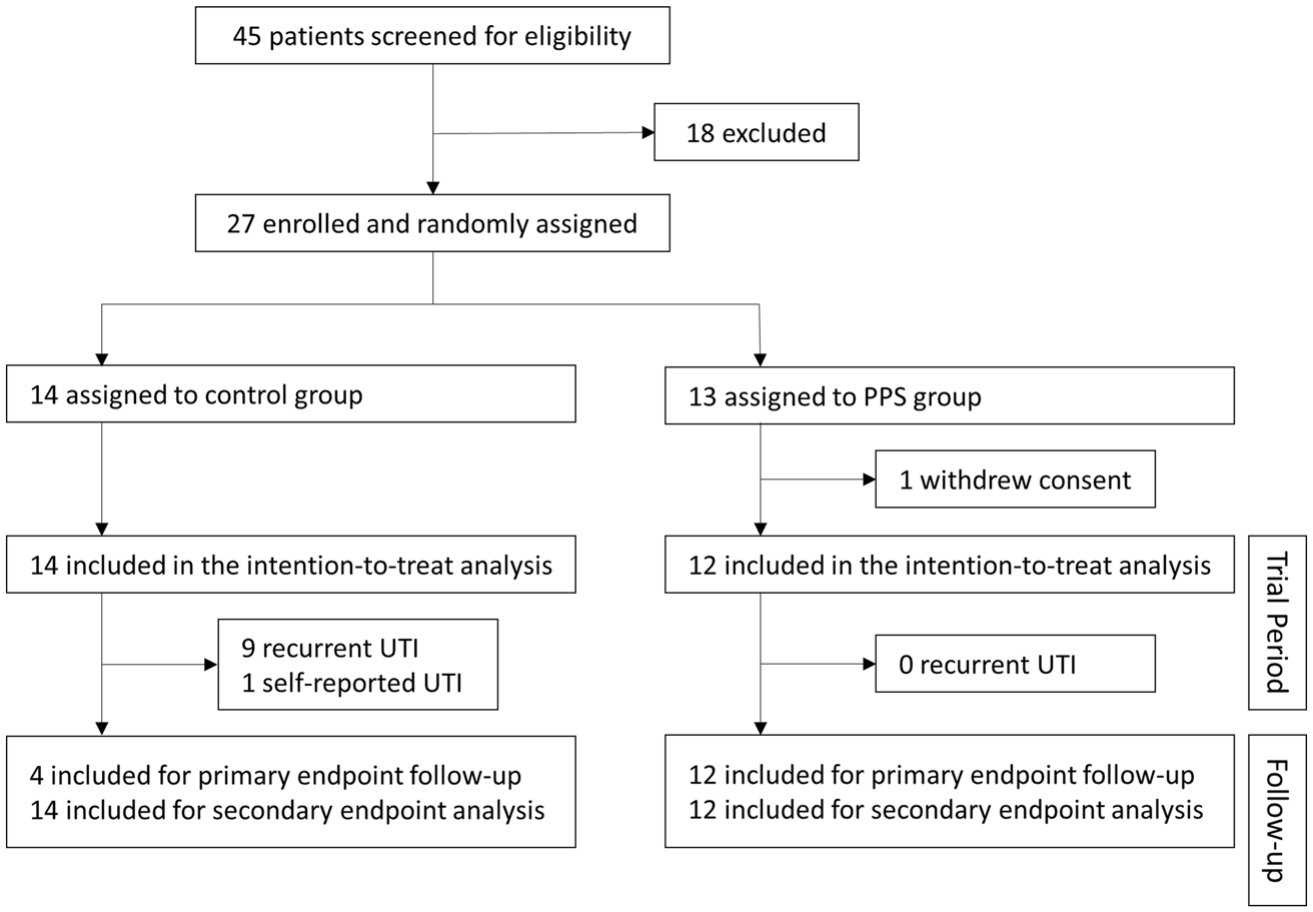
Study profile.

In this multicenter, open-label, phase 2, randomized controlled trial, women with recurrent UTI were treated with oral PPS or observation. Between June 2018 to May 2019, a total of 27 women meeting the inclusion criteria who gave written informed consent were enrolled and underwent randomization (Fig. 1). During the trial period, fourteen participants in the control group and twelve participants in the PPS group were followed for the occurrence of UTI events, which were determined and evaluated by board-certified urologists. After the completion of the trial, the trial participants were followed by their primary care physicians. Final surveys for the post-trial follow-up were conducted between July 2020 to January 2021, approximately two years after randomization for each participant. The sponsor was not involved in the collection or analysis of the data.

### Participants and procedures

Trial entry criteria included 20–70 years old women with UTI ≥ 2 times in the past 6 months or ≥ 3 times in the past 12 months with prior antibiotic treatment. Participants who had active UTI at screening were excluded. Participants were randomly assigned in a 1:1 ratio to receive either PPS monotherapy or observation according to the investigational site. Clinicians and participants were not masked to the allocation of treatment.

In the treatment group, PPS monotherapy was administered with an initial dose of 100 mg three times daily (300 mg/day) for 8 weeks and then with a maintenance dose of 100 mg twice daily (200 mg/day) for the following 8 weeks. In the post-trial period, PPS monotherapy was discontinued without any following prophylaxis for UTI in all patients. Treatment and follow-up for patients with recurrent UTI were under a consultant‐led appointment system with a patient-initiated system of care.

During the trial period, a diagnosis of UTI was made by a positive urine culture (defined as ≥ 100,000 CFU/mL by midstream urine)^12^, along with at least two lower urinary tract symptoms (such as dysuria, suprapubic pain, nocturia, frequency, urgency, or hematuria) or at least one specific result (as WBC > 5/HPF, nitrite positive, or leukocyte esterase positive) from urine analysis^13^.

### Post-trial data collection

The post-trial data collection was conducted approximately two years after randomization for each participant through phone interviews amid the pandemic of severe acute respiratory syndrome coronavirus 2 diseases. For safety assessment, patients were actively asked to report any adverse events that occurred after completion of PPS treatment during the post-trial period. Severe adverse events would be recorded as any life-threatening event, in-patient admission to hospital, or important medical event. For monitoring long-term protective effects, the patients who remained free from recurrent UTI at the end of the trial in both groups were asked to report any recurrent UTI episodes. For patients with UTI recurrence, the date of the first UTI episode, the types of lower urinary tract symptoms, and the number of overall recurrent UTI episodes during the post-trial period would be recorded.

The general QoL reflecting overall health status was assessed before trial entry and at post-trial follow-up survey. The score was subjectively selected by participants based on a 5-point scale, with 100 representing the participant's best imaginable health state and 0 representing the worst imaginable health state. A questionnaire of EQ-5D describing 5-dimensional health status was assessed along with QoL, including mobility, self-care, usual activities, pain/discomfort, and anxiety/depression^14,15^. Each dimension has three levels. Subjects were given three points for having no problems, two points for some problems, and one point for those who felt extremely problematic.

### Study outcomes

The primary endpoint was UTI recurrence-free survival, defined as the time from randomization to a documented UTI event or censoring at the date of the last follow-up. In the post-trial period, the diagnosis of UTI was made by at least two self-reported lower urinary tract symptoms by participants clinically. Microbiological confirmation for recurrent UTI was not mandatory. The secondary analyses assessed the satisfaction and well-being of participants before and approximately two years after the trial. Secondary endpoints included self-reported adverse events and QoL assessment by QoL scores and EQ-5D scores.

### Statistical analysis

Endpoints analyses were done in the intention-to-treat (ITT) population. Follow-up was calculated by the standard Kaplan–Meier method for the construction of UTI-free curves. A log-rank test was used for the comparison of recurrence-free survival duration between groups. The difference in QoL and EQ-5D changes between 0 and 24 months were tested for normality with Kolmogorov–Smirnov test and skewness with Kurtosis, and statistical inference with Student’s two-sample *t*-test. The difference between groups was done with Mann–Whitney *U* test and Student’s *t*-test for comparison of medians and means, respectively. All tests were 2-tailed and a *p* value of < 0.05 was considered significant. Statistical analyses were performed using the software: SPSS (version 25.0; IBM Corp, SPSS, Inc, Chicago, IL, USA) and PRISM program (GraphPad, V8.0.1).

### Ethical approval

The IRB of National Taiwan University Hospital (IRB# 201802019MSC), Taipei Tzu Chi Hospital (IRB# 07-FS01-013), and Tri-Service General Hospital (IRB# 1-106-01-035) all approved this study. All the procedures involving human participants followed in the study were in accordance with the ethical standards of the institutional and national research committee and with the 1964 Helsinki declaration.

## Results

Between June 2018 and May 2019, 27 patients were enrolled and randomly assigned to the control group (n = 14) or the PPS group (n = 13) for the original U101 study (Fig. 1). The efficacy of PPS monotherapy in UTI prevention was significantly superior to the control group which led to prompted termination of patient enrollment in this phase 2 trial^11^. One patient in the PPS group withdrew consent and resulted in 26 patients included in the ITT analysis and follow-up investigation for primary and secondary outcomes at approximately two years after randomization. The overall median follow-up duration was 25.8 months (IQR 24.8–27.6). Baseline characteristics including age, frequency of UTI before screening, renal function, and underlying medical conditions were all similar between the control group and the PPS group (Table 1). The median compliance rate of 16-week PPS monotherapy was 99.1% according to our prior report^11^.

**Table 1:** Demographic, clinical characteristics and outcomes of patients with recurrent UTI.

	Control group (n = 14)		PPS group (n = 12)		*P* value
Age (years)	57.7	8.54	51.1	8.9	0.067
UTI times before screening*	5.7	3.6	5.4	2.5	0.71
Serum creatinie (mg/dL)	0.63	0.07	0.71	0.13	0.062
**Medical history**					
Diabetes mellitus	0	0	0	0	–
Hypertension	1	7.10%	1	8.30%	0.91
Menopause	8	57.10%	5	41.70%	0.44
UTI recurrence within the trial (16 weeks)	9	64.3%	0	0.0%	< 0.001
UTI recurrence 1 year after trial	9	64.3%	2	16.7%	< 0.001
UTI recurrence for 24-month follow-up	12	85.7%	3	25.0%	< 0.001
**Quality of life**					
0 month (baseline)	80.7	4.3	76.7	12.1	0.254
24 months	77.5	5.5	91.7	7.5	< 0.001
Change from 0 to 24 months	− 3.2	5.4	15	15.3	< 0.001
**EQ-5D**					
0 month (baseline)	13.4	1.3	14.2	1.1	0.101
24 months	13.3	1.3	14.8	0.5	0.001
Change from 0 to 24 months	− 0.1	1.1	0.6	1.2	0.174

Data are mean (SD); or n (%). *Statistical analyses were carried out using Student’s t-test to compare means between the patient groups.

### UTI recurrence-free survival

**Figure 2:**
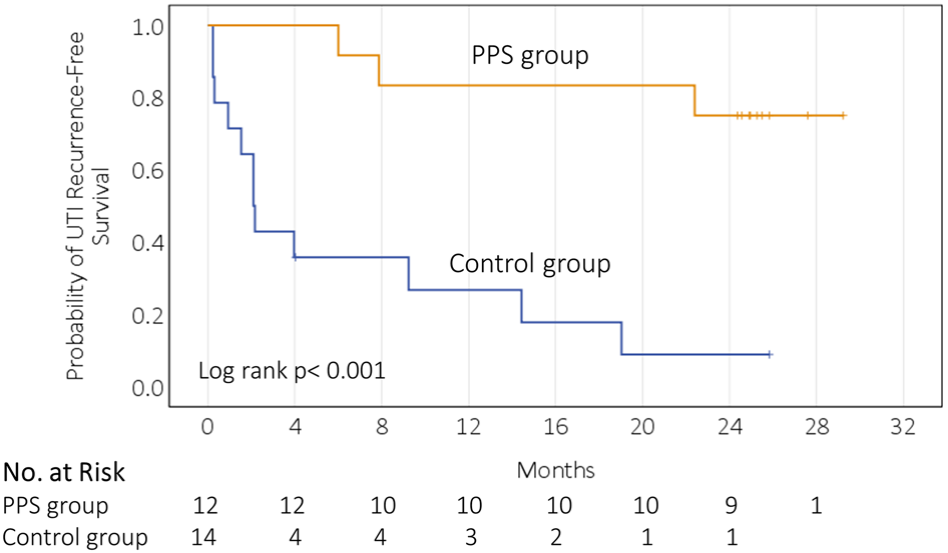
Kaplan–Meier survival curves for UTI recurrence-free survival in 26 patients.

The median duration of follow-up for recurrent UTI in the PPS group was 24.9 months (IQR 22.9–25.8). Only three out of twelve participants (25%) had self-reported UTI recurrence within 24 months during the follow-up period (Table 1). In contrast, twelve out of fourteen participants (85.7%) in the control group had UTI recurrence during the in-trial (9 recurrent UTI cases) and the follow-up (3 recurrent UTI cases) period. The UTI recurrence-free survival (Fig. 2) remained significantly higher in the PPS group than in the control group during the in-trial and follow-up period (log-rank test *p* < 0.001). The hazard ratio for UTI recurrence-free survival was 0.11 (95% confidence interval [CI], 0.03–0.43) for the comparison of the PPS group with the control group.

Aside from one serious adverse event of temporary hearing loss which resolved completely after steroid treatment during the trial period, no other drug-related adverse event was reported during the 2-year follow-up period.

### Quality of life

**Figure 3:**
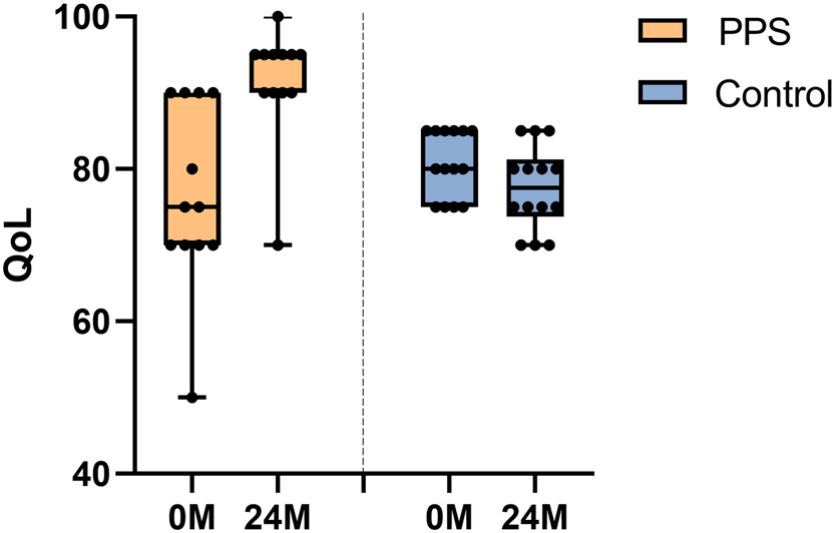
Changes in quality of life score between 0 M (before trial) and 24 M (2 years after trial initiation) in each group. Group mean is shown with a line, and whiskers represent the min to max.

QoL score at 0 month in the PPS group was normally distributed (Kolmogorov–Smirnov test *p* = 0.16) and without skewness (Z = − 1.02). The mean QoL score at 24 months had increased by 15.0 points (*p* = 0.006) compared to 0 month before treatment in the PPS group (Table 1, Fig. 3). However, the mean QoL score in the control group had decreased by 3.2 points at 24 months (*p* = 0.045). For comparison between the two groups, the QoL score was significantly higher in the PPS group than the control group at 24 months (*p* < 0.001) but not at 0 month (*p* = 0.254) (Table 1).

**Figure 4:**
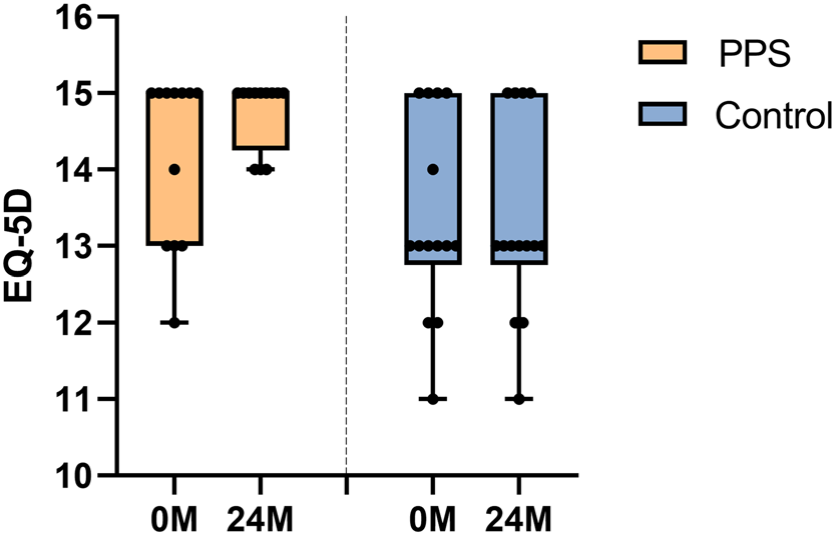
Changes in EQ-5D total score between 0 M (before trial) and 24 M (2 years after trial initiation) in each group. Group mean is shown with a line, and whiskers represent the min to max.

The ITT analysis for EQ-5D-3L score did not show a significant difference between groups (*p* = 0.101) at baseline 0-month (Table 1). For EQ-5D score at 24 months, the PPS group was significantly higher than control group (*p* = 0.001). EQ-5D score showed an improvement in the PPS group of 0.6 points (95% CI, − 0.2–1.4; *p* = 0.131) (Fig. 4). In contrast, the change of EQ-5D from baseline 0 month to 24 months was − 0.1 points (95% CI, − 0.7–0.6; *p* = 0.818).

**Figure 5:**
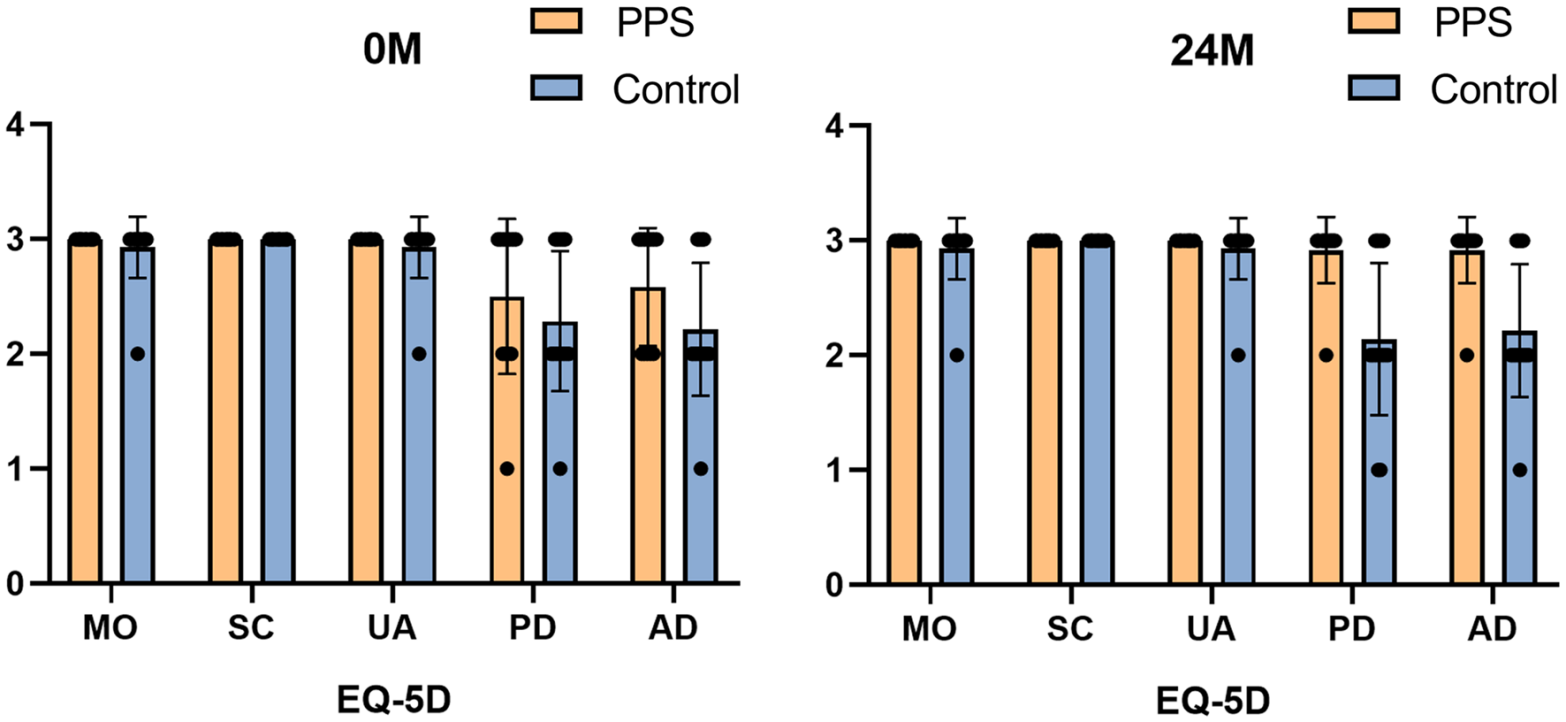
Scores in each domain of EQ-5D between PPS and control group at 0 M and 24 M. MO indicates mobility; SC, self-care; UA, usual activities; PD, pain/discomfort; and AD, anxiety/depression.

Scores in each dimension of EQ-5D among PPS and control group at 0 month to 24 months were shown in Fig. 5 and Table 2. EQ-5D subscores at 24 months were significantly higher in the PPS group than control group for the pain/discomfort (*p* = 0.001) and anxiety/depression (*p* = 0.004) scores. No other subscores for each domain between two groups reached statistical significance at baseline 0 month or 24 months. The changes in EQ-5D-3L didn’t show a significant difference between the two groups.

**Table 2:** Comparison of the EQ-5D-3L scores by domain between two groups.

	Control group		PPS group		*p* value
Number of patients	14		12		
**0 month (baseline)**					
EQ-5D	13.4	1.3	14.2	1.1	0.101
Mobility (MO)	2.9	0.3	3	0	0.365
Self-care (SC)	3	0	3	0	–
Usual activities (UA)	2.9	0.3	3	0	0.365
Pain/discomfort (PD)	2.3	0.6	2.6	0.7	0.247
Anxiety/depression (AD)	2.2	0.6	2.6	0.1	0.101
**24 months**					
EQ-5D	13.3	1.3	14.8	0.5	0.001
Mobility (MO)	2.9	0.3	3	0	0.365
Self-care (SC)	3	0	3	0	–
Usual activites (UA)	2.9	0.3	3	0	0.365
Pain/discomfort (PD)	2.1	0.7	2.9	0.3	0.001
Anxiety/depression (AD)	2.2	0.6	2.8	0.4	0.004
**Change from 0 to 24 months**					
EQ-5D	− 0.1	1.1	0.6	1.2	0.174
Mobility (MO)	0	0	0	0	–
Self-care (SC)	0	0	0	0	–
Usual activities (UA)	0	0	0	0	–
Pain/discomfort (PD)	− 0.1	0.7	0.3	0.8	0.289
Anxiety/depression (AD)	0	0.6	0.3	0.6	0.294

Data are mean (SD); or n (%). *Statistical analyses were carried out using Student’s t-test to compare means between the patient groups.

## Discussion

The follow-up results of the multicenter, open-label, prospective, phase 2a, randomized controlled trial demonstrated that 16-week PPS monotherapy significantly improved UTI recurrence-free survival for 2 years compared with the control arm. In this report, the recurrent rate in one year after the study was 16.7% (2/12 participants) in the PPS group and 64.3% (9/14 participants) in the control group. To our knowledge, this was the first clinical trial using PPS monotherapy for recurrent UTI prevention which showed promising results in both trial period and post-trial follow-up period.

Continuous antibiotic prophylaxis was the most efficacious method in preventing recurrent UTIs in the literature^8–10^. Antibiotic prophylaxis for 6 to 12 months reduced the risk of recurrence in women with recurrent UTI (at least 2 episodes in 12 months) to 12.3% compared with the recurrence rate of 65.5% in the placebo group^16^. Comparatively, antibiotics represented higher severe side effects with a relative risk of 1.58 (95% CI 0.47–5.28) than the placebo group. Besides, antibiotic prophylaxis loses the power of protection soon after discontinuation of the treatment^16^. In the 6 months follow-up period after an antimicrobial prophylaxis study, the recurrence rate in the antibiotic group and placebo-controlled group became identical^17^.

AUA/CUA/SUFU guidelines indicate cranberry prophylaxis for recurrent UTIs as a conditional recommendation with the evidence level Grade C^7^. The possible mechanism is the proanthocyanidins in cranberries which can inhibit the adhesion of P-fimbriated E. coli to the urothelial cell receptor. Cranberry oral juice or tablet supplements might be offered according to availability and patients’ preference. A trial comparing two treatments, cranberry and lactobacillus, against the placebo group showed that at six months, 16% of women in the cranberry group, 39% in the lactobacillus group, and 36% in the control group had UTI recurrence for at least one episode^18^. However, in a comparative trial for five prevention strategies targeting patients with eight or more UTIs a year, an average of 4.4 recurrent episodes in a year occurred in the cranberry prophylaxis group, which was higher than 3.1 episodes in the daily estrogen group, 2.8 episodes per year in the acupuncture group, and 1.3 episodes in the antimicrobial prophylaxis group^9^. In another trial of 319 college women, the active cranberry prophylaxis for six months presented a higher recurrence rate of UTI by 20% versus 14% in the control group^10^. Nevertheless, cranberry prophylaxis is still recommended because of the safety profile of the food-based supplement.

Vaginal hormonal replacement is unanimously recommended by several clinical practice guidelines for peri- and post-menopausal women with recurrent UTIs^19^. However, several other agents for UTI prevention revealed controversial results of their efficacy^7^. The panel of AUA/CUA/SUFU guidelines could not find sufficient evidence to recommend other prevention methods such as lactobacillus, D-mannose, methenamine, herbal supplements, intravesical instillation of hyaluronic acid or chondroitin, and immunoactive prophylaxis^7^.

Methylamine salts appeared to be safe and effective as an alternative to antibiotics for the prevention of UTI in the literature^20^. In a randomized non-inferiority trial for UTI prevention, methenamine hippurate was as effective as low-dose antibiotics for women with a history of recurrent UTI^21^. However, nearly 56% of patients using methenamine hippurate still received therapeutic antibiotics for UTI during the 12-month treatment period. During the 6-month post-trial follow-up period, UTI rates and duration of antibiotic use were found to be higher in the methenamine hippurate group than in the low-dose antibiotic prophylaxis group.

Glycosaminoglycan (GAG) replacement therapy, hyaluronic acid (HA) with or without chondroitin sulfate intravesical instillation, is thought to be promising in treating recurrent UTI in previous studies^22–24^. The treatment aims to restore the important lining of urothelial epithelium which plays an important role in stopping bacterial invasion and adherence^25,26^. With the same concept, heparin intravesical instillation was studied for treating interstitial cystitis^27^. GAG replacement therapy demonstrated a durable protection effect for free from recurrent UTI for up to 47 weeks after 9 times of HA instillations over 6 months^22^. Other research revealed a less satisfactory result that only 48% of patients were free from UTI in 12 months after a combination intravesical instillation of HA and chondroitin sulfate^23^. Because of the high cost of HA and chondroitin sulfate and the requirement of catheterization for each bladder instillation, the treatment is unattainable in real-world practice.

PPS, a sulfated polysaccharide, was shown to be effective at restoring GAG-deficient bladder mucosa^28^ and binding to urothelium with sufficient strength against bladder washing^29^ in animal models. For patients with painful bladder syndrome/interstitial cystitis, the use of oral PPS was able to improve symptoms and repair abnormal bladder surface glycosaminoglycans^30^. Therefore, the oral administration of PPS brought great convenience to both clinicians and patients considering GAG replacement therapy with high compliance and long-term protective effect.

A qualitative analysis revealed that the patient attitudes toward antibiotic management of recurrent UTIs are dominated by fear and frustration^5^. Women reported fear of unnecessary antibiotics and the development of antimicrobial resistance as well. Patients even reported frustrations with the limited effect of nonantibiotic prophylaxis strategies for recurrent UTIs. However, effective alternative prophylaxis, using oral immunostimulation with bacterial extracts of E. coli, could significantly improve the QoL of patients, including the reduction in anxiety and depression^31^. In this 2-year follow-up study, recurrent UTIs had an unfavorable effect on general QoL whereas QoL improvement was observed at 24 months after PPS treatment. In terms of EQ-5D scores, patients without PPS prophylaxis showed significantly lower scores than the PPS group in two dimensions, representing physical pain/discomfort and mental anxiety/depression. Other dimensions, including mobility, self-care, and usual activities, were not influenced by UTI before trial entry or at the 24-month follow-up.

The limitations of this phase 2a follow-up study were the small number of cases and lack of a placebo control arm. Therefore, a phase 3 double-blind, randomized, placebo-controlled trial has been launched to verify the results of this phase 2a study. During the follow-up period, the diagnostic criteria for UTIs were loose, without confirmation by positive urine culture, which might overestimate the number of UTI events. Nevertheless, the results still highlighted the fact that PPS monotherapy has a long-term effect in preventing recurrent UTI.

## Conclusions

The follow-up study showed that a 16-week oral PPS monotherapy could prevent uncomplicated recurrent UTIs up to 24 months after cessation of PPS treatment. We identified a significant improvement in overall QoL in the PPS group. The ongoing phase 3 trial of PPS monotherapy is expected to provide more solid evidence for this promising prophylaxis agent.

## Data Availability

The datasets used and/or analysed during the current study available from the corresponding author on reasonable request.

## Abbreviations

UTI: Urinary tract infection
PPS: Pentosan polysulfate sodium
ITT: Intention-to-treat
EQ-5D: EuroQol five-dimensional
